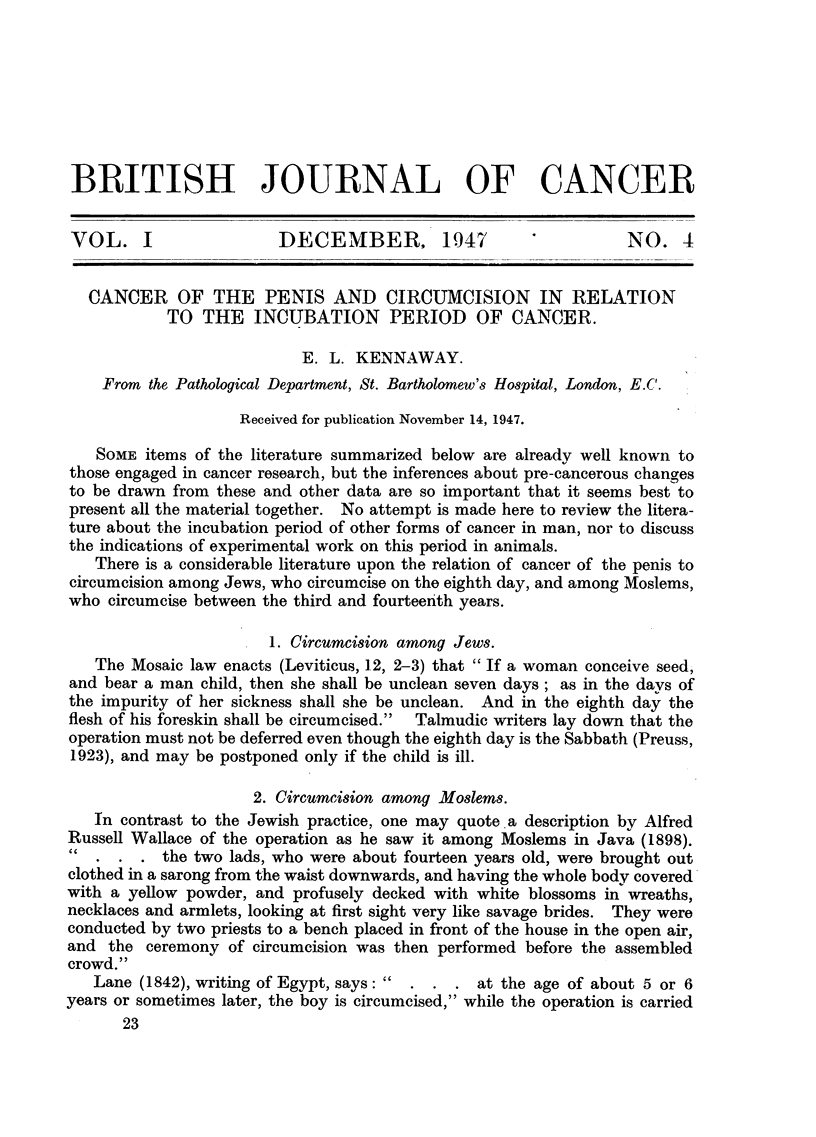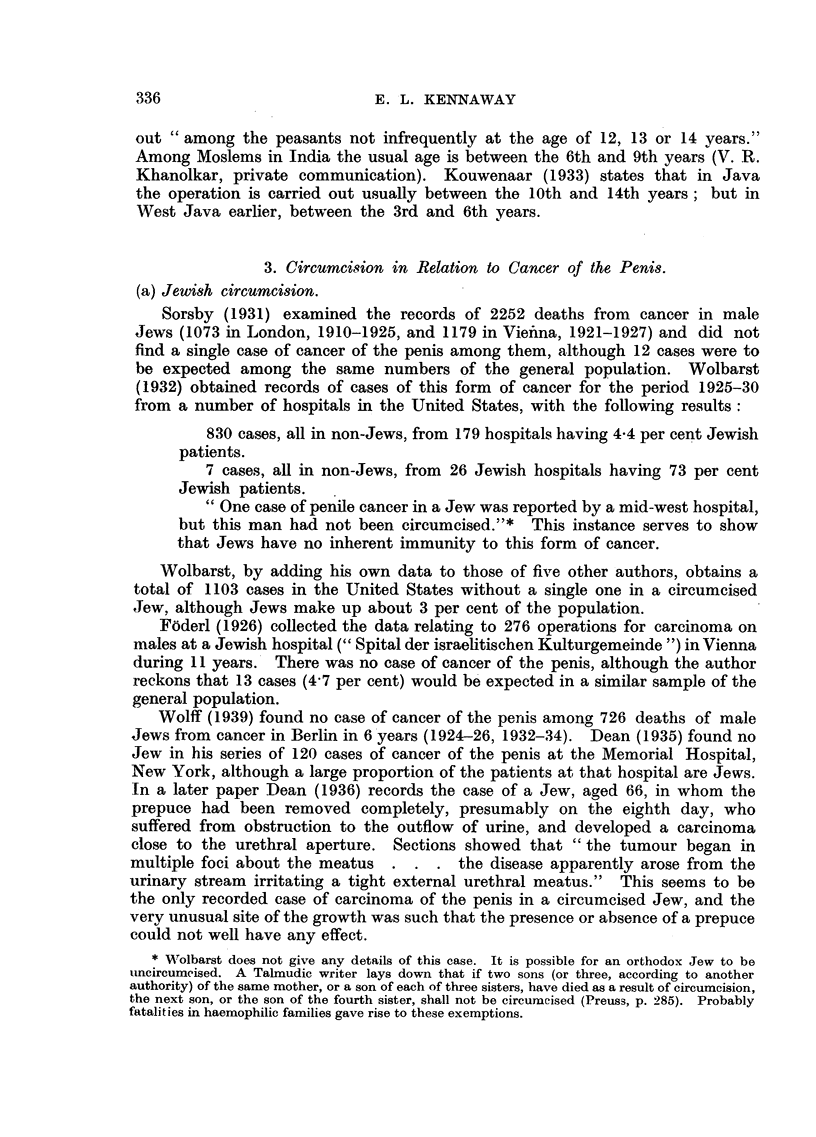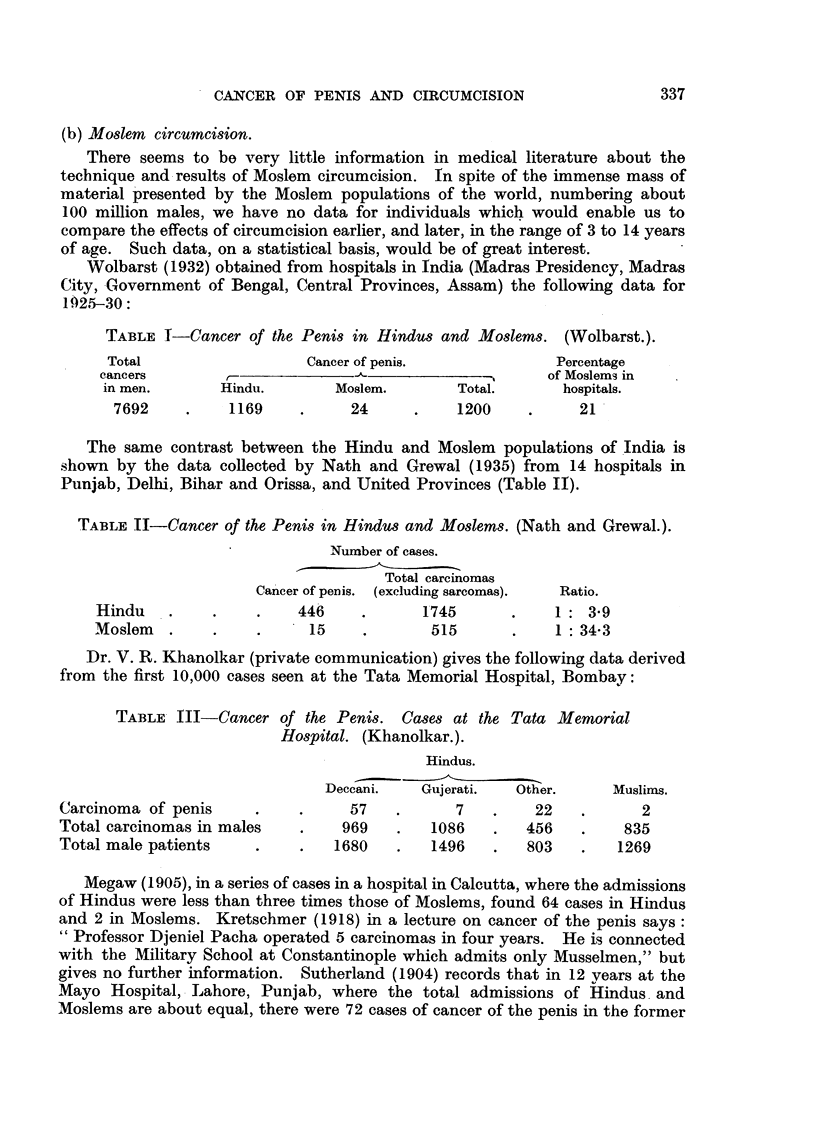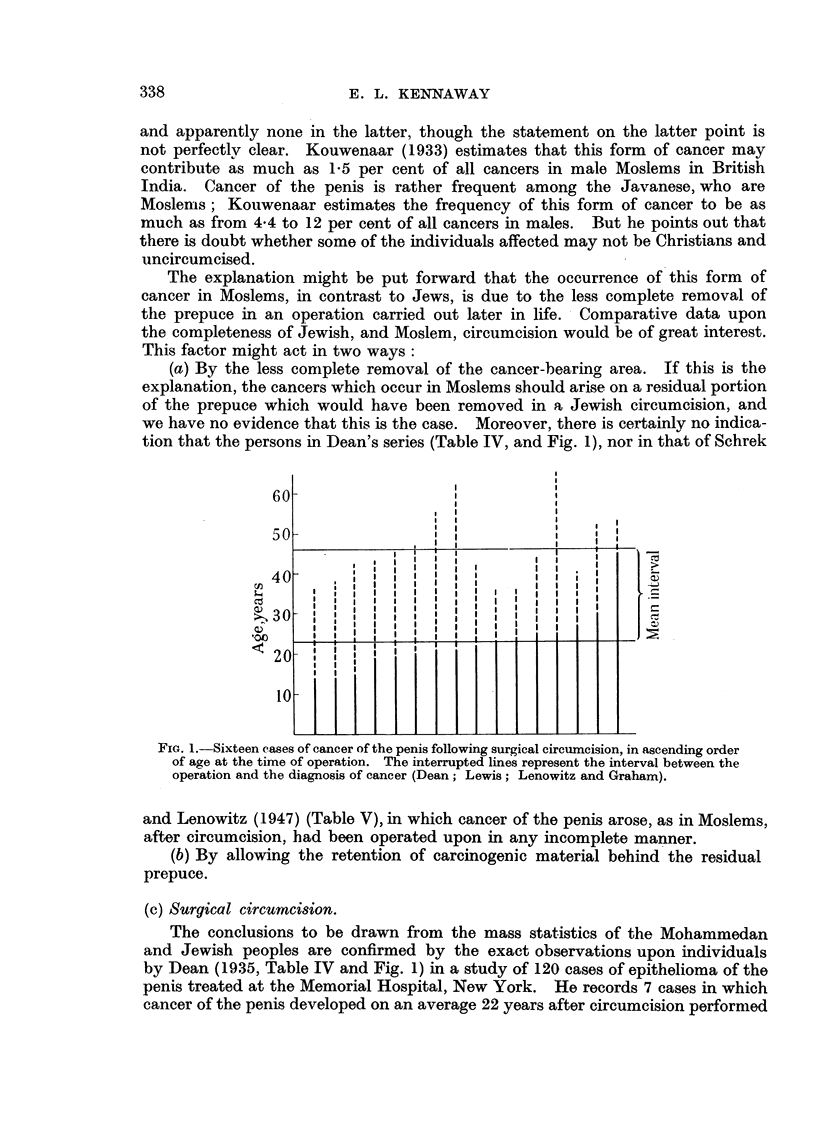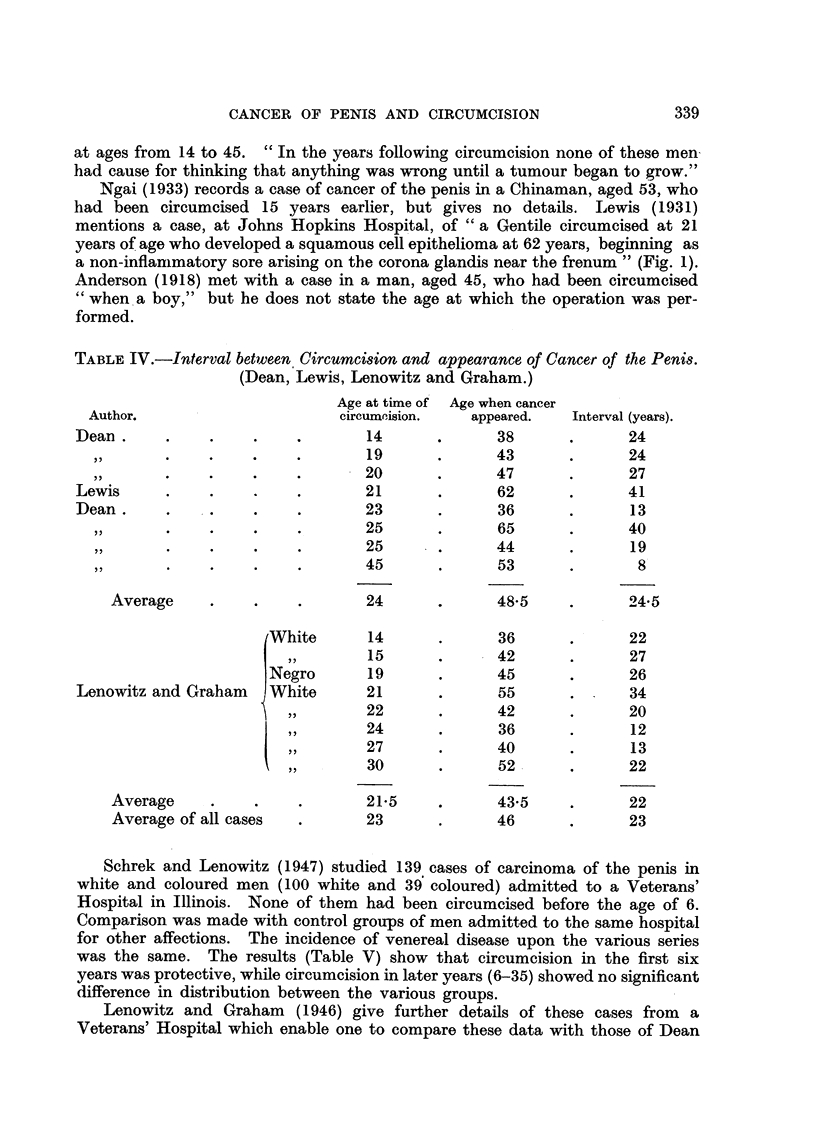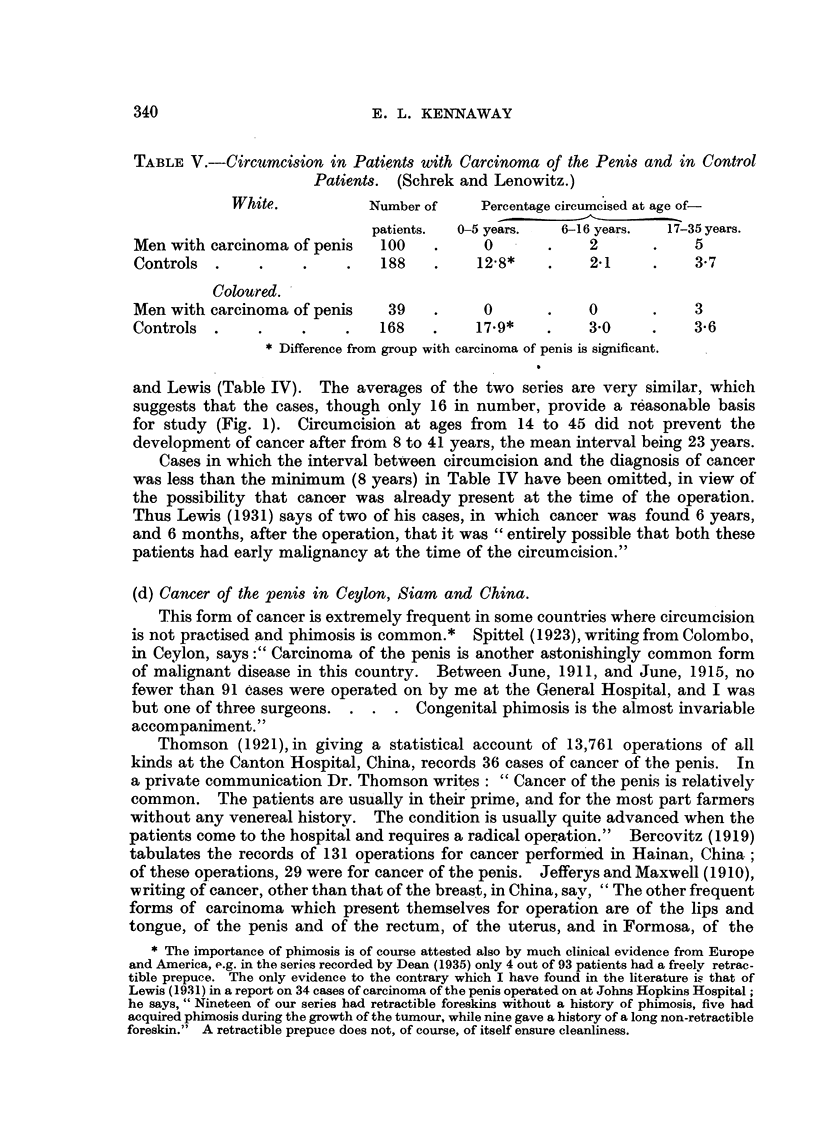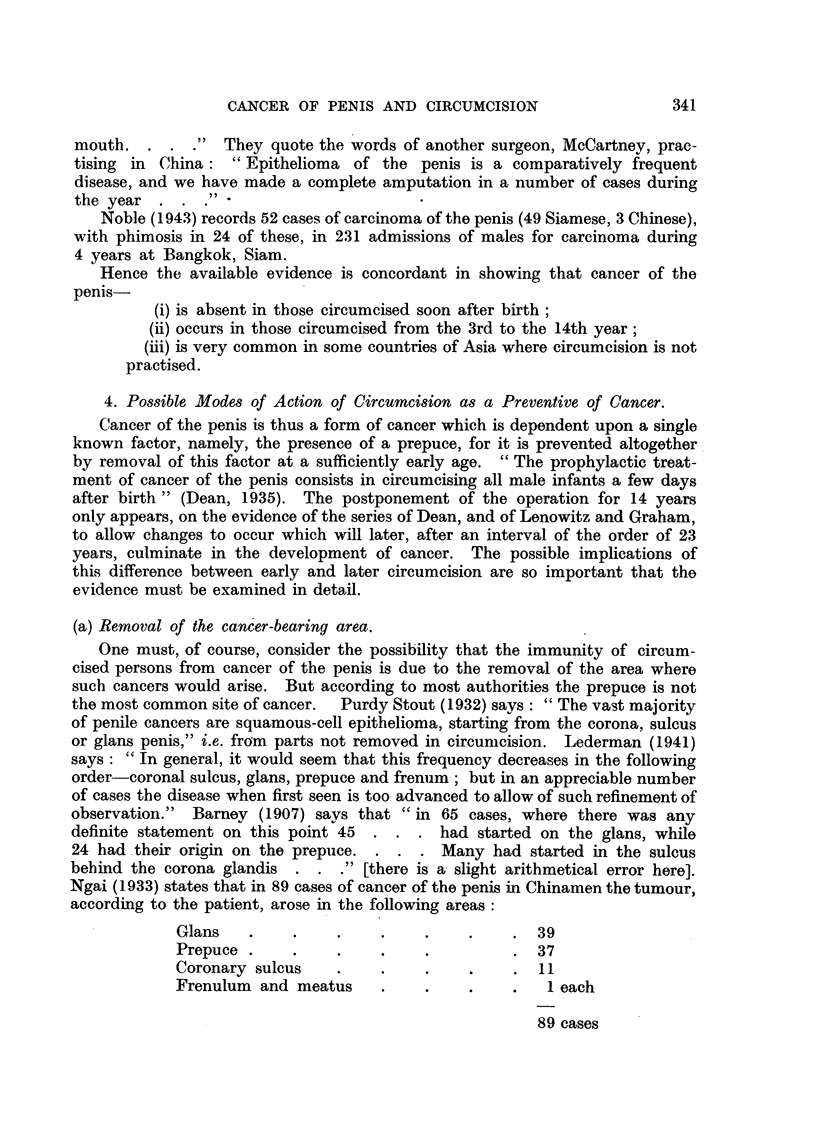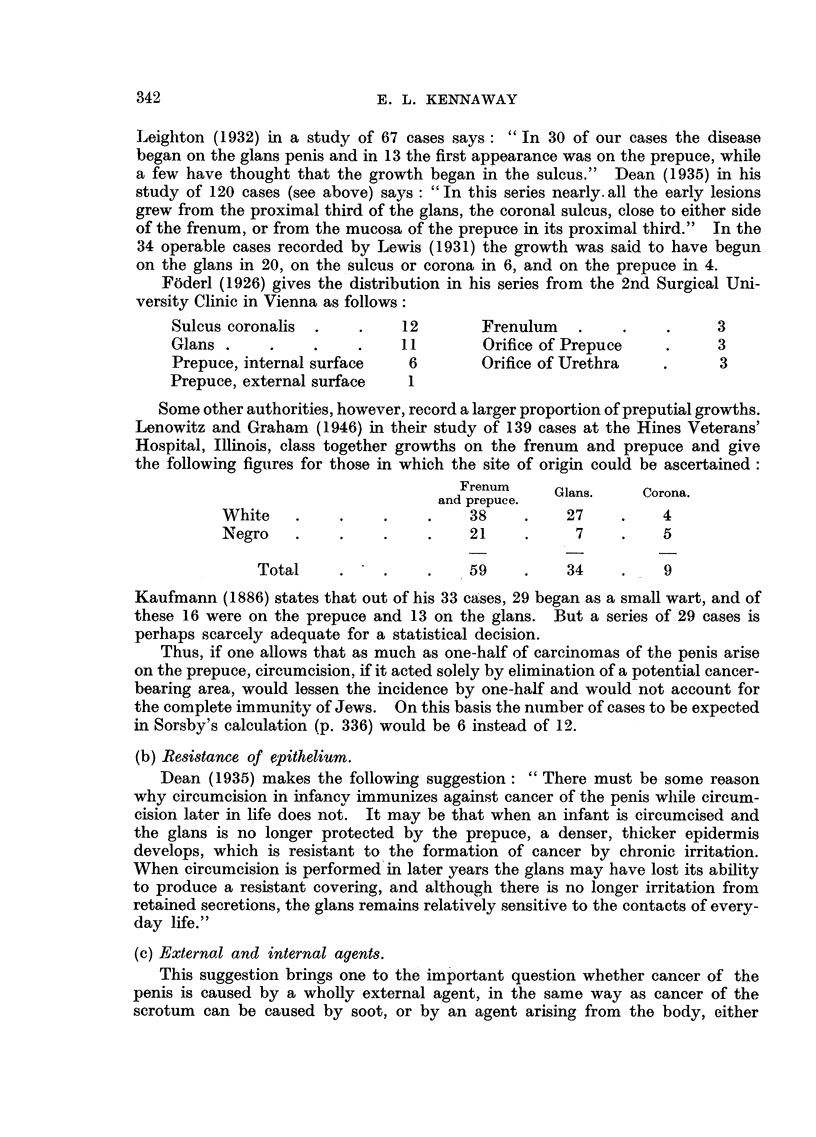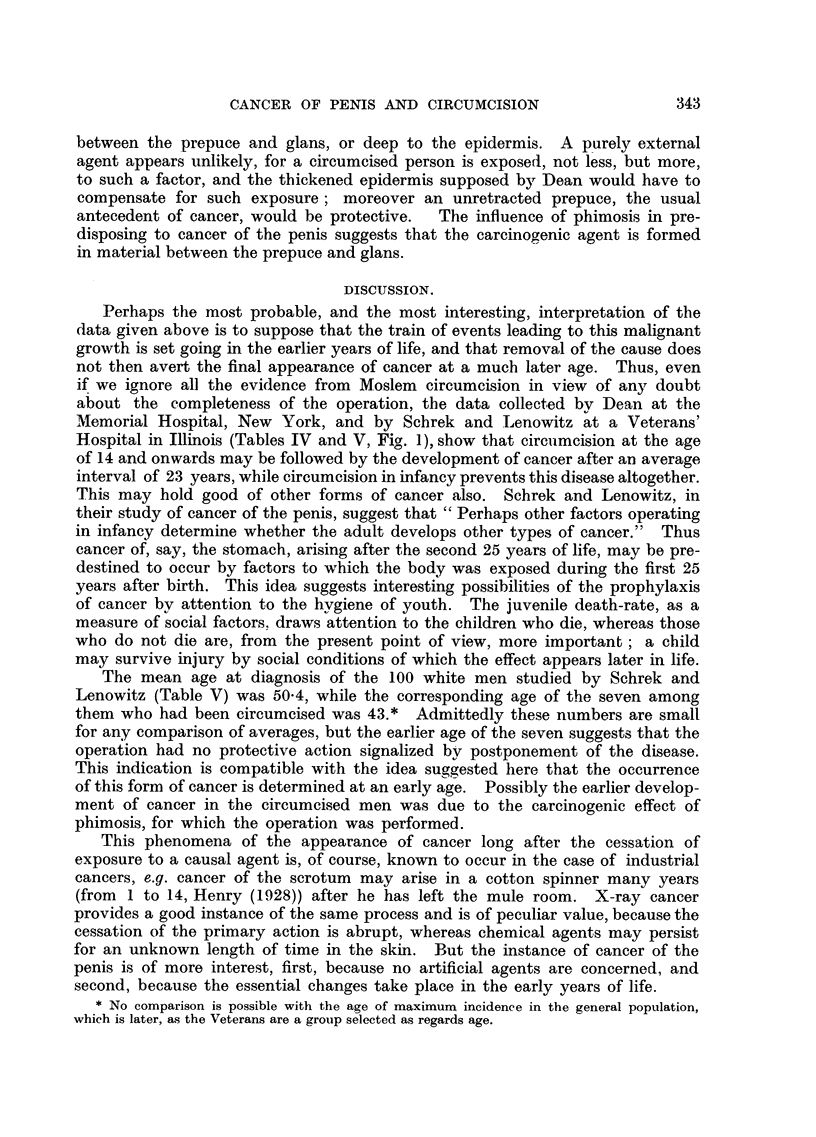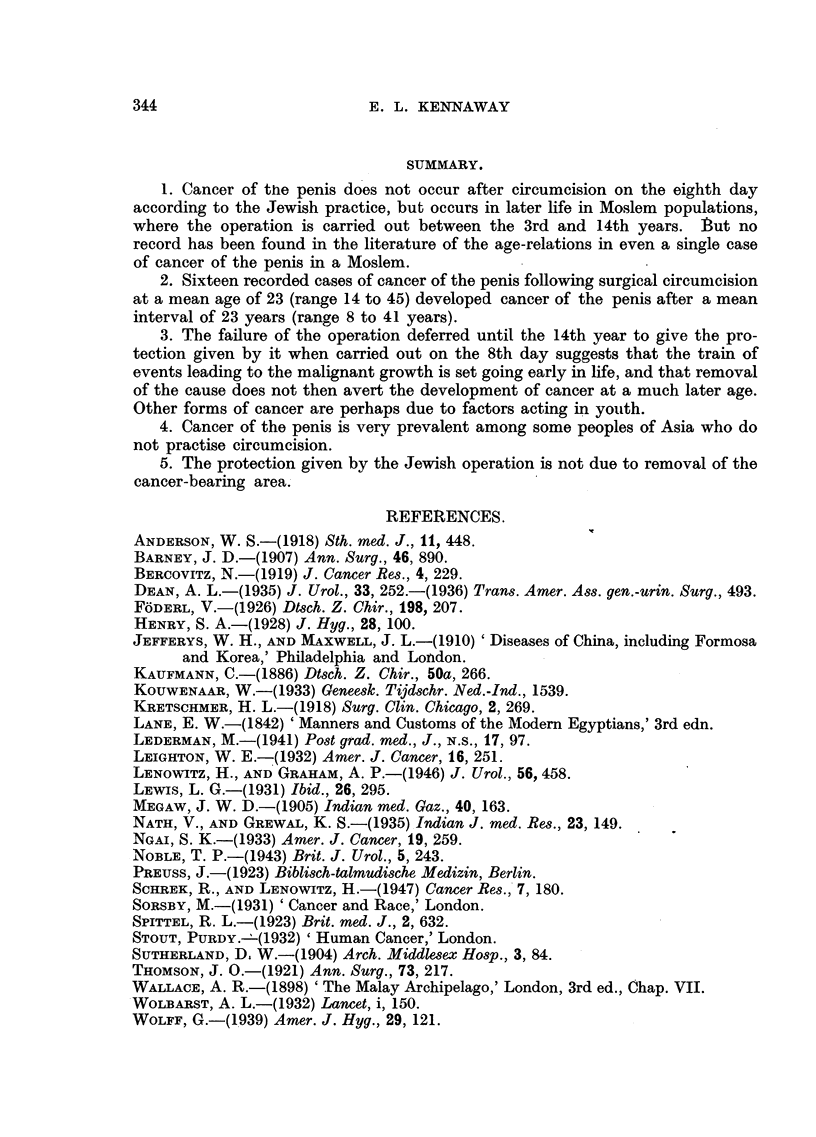# Cancer of the Penis and Circumcision in Relation to the Incubation Period of Cancer

**DOI:** 10.1038/bjc.1947.30

**Published:** 1947-12

**Authors:** E. L. Kennaway


### BRITISH JOURNAL OF CANCER

VOL. I DECEMBER, 1947 NO. 4

### CANCER OF THE PENIS AND CIRCUMCISION IN RELATION TO THE INCUBATION PERIOD OF CANCER.

E. L. KENNAWAY.

*From the Pathological Department, St. Bartholomew's Hospital, London, E.C.*

Received for publication November 14, 1947.

SOME items of the literature summarized below are already well known to those engaged in cancer research, but the inferences about pre-cancerous changes to be drawn from these and other data are so important that it seems best to present all the material together. No attempt is made here to review the literature about the incubation period of other forms of cancer in man, nor to discuss the indications of experimental work on this period in animals.

There is a considerable literature upon the relation of cancer of the penis to circumcision among Jews, who circumcise on the eighth day, and among Moslems, who circumcise between the third and fourteenth years.

### 1. *Circumcision among Jews.*

The Mosaic law enacts (Leviticus, 12, 2–3) that "If a woman conceive seed, and bear a man child, then she shall be unclean seven days ; as in the days of the impurity of her sickness shall she be unclean. And in the eighth day the flesh of his foreskin shall be circumcised." Talmudic writers lay down that the operation must not be deferred even though the eighth day is the Sabbath (Preuss, 1923), and may be postponed only if the child is ill.

### 2. *Circumcision among Moslems.*

In contrast to the Jewish practice, one may quote a description by Alfred Russell Wallace of the operation as he saw it among Moslems in Java (1898). " . . . the two lads, who were about fourteen years old, were brought out clothed in a sarong from the waist downwards, and having the whole body covered with a yellow powder, and profusely decked with white blossoms in wreaths, necklaces and armlets, looking at first sight very like savage brides. They were conducted by two priests to a bench placed in front of the house in the open air, and the ceremony of circumcision was then performed before the assembled crowd."

Lane (1842), writing of Egypt, says : " . . . at the age of about 5 or 6 years or sometimes later, the boy is circumcised," while the operation is carried out " among the peasants not infrequently at the age of 12, 13 or 14 years." Among Moslems in India the usual age is between the 6th and 9th years (V. R. Khanolkar, private communication). Kouwenaar (1933) states that in Java the operation is carried out usually between the 10th and 14th years; but in West Java earlier, between the 3rd and 6th years.

### 3. *Circumcision in Relation to Cancer of the Penis.*

(a) *Jewish circumcision.*

Sorsby (1931) examined the records of 2252 deaths from cancer in male Jews (1073 in London, 1910–1925, and 1179 in Vienna, 1921–1927) and did not find a single case of cancer of the penis among them, although 12 cases were to be expected among the same numbers of the general population. Wolbarst (1932) obtained records of cases of this form of cancer for the period 1925–30 from a number of hospitals in the United States, with the following results:

> 830 cases, all in non-Jews, from 179 hospitals having 4·4 per cent Jewish patients.
>
> 7 cases, all in non-Jews, from 26 Jewish hospitals having 73 per cent Jewish patients.
>
> " One case of penile cancer in a Jew was reported by a mid-west hospital, but this man had not been circumcised."* This instance serves to show that Jews have no inherent immunity to this form of cancer.

Wolbarst, by adding his own data to those of five other authors, obtains a total of 1103 cases in the United States without a single one in a circumcised Jew, although Jews make up about 3 per cent of the population.

Föderl (1926) collected the data relating to 276 operations for carcinoma on males at a Jewish hospital (" Spital der israelitischen Kulturgemeinde ") in Vienna during 11 years. There was no case of cancer of the penis, although the author reckons that 13 cases (4·7 per cent) would be expected in a similar sample of the general population.

Wolff (1939) found no case of cancer of the penis among 726 deaths of male Jews from cancer in Berlin in 6 years (1924–26, 1932–34). Dean (1935) found no Jew in his series of 120 cases of cancer of the penis at the Memorial Hospital, New York, although a large proportion of the patients at that hospital are Jews. In a later paper Dean (1936) records the case of a Jew, aged 66, in whom the prepuce had been removed completely, presumably on the eighth day, who suffered from obstruction to the outflow of urine, and developed a carcinoma close to the urethral aperture. Sections showed that " the tumour began in multiple foci about the meatus . . . the disease apparently arose from the urinary stream irritating a tight external urethral meatus." This seems to be the only recorded case of carcinoma of the penis in a circumcised Jew, and the very unusual site of the growth was such that the presence or absence of a prepuce could not well have any effect.

* Wolbarst does not give any details of this case. It is possible for an orthodox Jew to be uncircumcised. A Talmudic writer lays down that if two sons (or three, according to another authority) of the same mother, or a son of each of three sisters, have died as a result of circumcision, the next son, or the son of the fourth sister, shall not be circumcised (Preuss, p. 285). Probably fatalities in haemophilic families gave rise to these exemptions.

(b) *Moslem circumcision.*

There seems to be very little information in medical literature about the technique and results of Moslem circumcision. In spite of the immense mass of material presented by the Moslem populations of the world, numbering about 100 million males, we have no data for individuals which would enable us to compare the effects of circumcision earlier, and later, in the range of 3 to 14 years of age. Such data, on a statistical basis, would be of great interest.

Wolbarst (1932) obtained from hospitals in India (Madras Presidency, Madras City, Government of Bengal, Central Provinces, Assam) the following data for 1925–30:

TABLE I—*Cancer of the Penis in Hindus and Moslems.* (Wolbarst.).

| Total cancers in men. | Cancer of penis. | | | Percentage of Moslems in hospitals. |
|---|---|---|---|---|
| | Hindu. | Moslem. | Total. | |
| 7692 | 1169 | 24 | 1200 | 21 |

The same contrast between the Hindu and Moslem populations of India is shown by the data collected by Nath and Grewal (1935) from 14 hospitals in Punjab, Delhi, Bihar and Orissa, and United Provinces (Table II).

TABLE II—*Cancer of the Penis in Hindus and Moslems.* (Nath and Grewal.).

| | Number of cases. | | |
|---|---|---|---|
| | Cancer of penis. | Total carcinomas (excluding sarcomas). | Ratio. |
| Hindu | 446 | 1745 | 1 : 3·9 |
| Moslem | 15 | 515 | 1 : 34·3 |

Dr. V. R. Khanolkar (private communication) gives the following data derived from the first 10,000 cases seen at the Tata Memorial Hospital, Bombay:

TABLE III—*Cancer of the Penis. Cases at the Tata Memorial Hospital.* (Khanolkar.).

| | Hindus. | | | |
|---|---|---|---|---|
| | Deccani. | Gujerati. | Other. | Muslims. |
| Carcinoma of penis | 57 | 7 | 22 | 2 |
| Total carcinomas in males | 969 | 1086 | 456 | 835 |
| Total male patients | 1680 | 1496 | 803 | 1269 |

Megaw (1905), in a series of cases in a hospital in Calcutta, where the admissions of Hindus were less than three times those of Moslems, found 64 cases in Hindus and 2 in Moslems. Kretschmer (1918) in a lecture on cancer of the penis says: "Professor Djeniel Pacha operated 5 carcinomas in four years. He is connected with the Military School at Constantinople which admits only Musselmen," but gives no further information. Sutherland (1904) records that in 12 years at the Mayo Hospital, Lahore, Punjab, where the total admissions of Hindus and Moslems are about equal, there were 72 cases of cancer of the penis in the former and apparently none in the latter, though the statement on the latter point is not perfectly clear. Kouwenaar (1933) estimates that this form of cancer may contribute as much as 1·5 per cent of all cancers in male Moslems in British India. Cancer of the penis is rather frequent among the Javanese, who are Moslems ; Kouwenaar estimates the frequency of this form of cancer to be as much as from 4·4 to 12 per cent of all cancers in males. But he points out that there is doubt whether some of the individuals affected may not be Christians and uncircumcised.

The explanation might be put forward that the occurrence of this form of cancer in Moslems, in contrast to Jews, is due to the less complete removal of the prepuce in an operation carried out later in life. Comparative data upon the completeness of Jewish, and Moslem, circumcision would be of great interest. This factor might act in two ways :

(*a*) By the less complete removal of the cancer-bearing area. If this is the explanation, the cancers which occur in Moslems should arise on a residual portion of the prepuce which would have been removed in a Jewish circumcision, and we have no evidence that this is the case. Moreover, there is certainly no indication that the persons in Dean's series (Table IV, and Fig. 1), nor in that of Schrek

Fig. 1.—Sixteen cases of cancer of the penis following surgical circumcision, in ascending order of age at the time of operation. The interrupted lines represent the interval between the operation and the diagnosis of cancer (Dean ; Lewis ; Lenowitz and Graham).

and Lenowitz (1947) (Table V), in which cancer of the penis arose, as in Moslems, after circumcision, had been operated upon in any incomplete manner.

(*b*) By allowing the retention of carcinogenic material behind the residual prepuce.

(c) *Surgical circumcision.*

The conclusions to be drawn from the mass statistics of the Mohammedan and Jewish peoples are confirmed by the exact observations upon individuals by Dean (1935, Table IV and Fig. 1) in a study of 120 cases of epithelioma of the penis treated at the Memorial Hospital, New York. He records 7 cases in which cancer of the penis developed on an average 22 years after circumcision performed at ages from 14 to 45. " In the years following circumcision none of these men had cause for thinking that anything was wrong until a tumour began to grow."

Ngai (1933) records a case of cancer of the penis in a Chinaman, aged 53, who had been circumcised 15 years earlier, but gives no details. Lewis (1931) mentions a case, at Johns Hopkins Hospital, of " a Gentile circumcised at 21 years of age who developed a squamous cell epithelioma at 62 years, beginning as a non-inflammatory sore arising on the corona glandis near the frenum " (Fig. 1). Anderson (1918) met with a case in a man, aged 45, who had been circumcised " when a boy," but he does not state the age at which the operation was performed.

TABLE IV.—*Interval between Circumcision and appearance of Cancer of the Penis.* (Dean, Lewis, Lenowitz and Graham.)

| Author. | | Age at time of circumcision. | Age when cancer appeared. | Interval (years). |
|---|---|---|---|---|
| Dean | | 14 | 38 | 24 |
| ,, | | 19 | 43 | 24 |
| ,, | | 20 | 47 | 27 |
| Lewis | | 21 | 62 | 41 |
| Dean | | 23 | 36 | 13 |
| ,, | | 25 | 65 | 40 |
| ,, | | 25 | 44 | 19 |
| ,, | | 45 | 53 | 8 |
| Average | | 24 | 48·5 | 24·5 |
| Lenowitz and Graham | White | 14 | 36 | 22 |
| | ,, | 15 | 42 | 27 |
| | Negro | 19 | 45 | 26 |
| | White | 21 | 55 | 34 |
| | ,, | 22 | 42 | 20 |
| | ,, | 24 | 36 | 12 |
| | ,, | 27 | 40 | 13 |
| | ,, | 30 | 52 | 22 |
| Average | | 21·5 | 43·5 | 22 |
| Average of all cases | | 23 | 46 | 23 |

Schrek and Lenowitz (1947) studied 139 cases of carcinoma of the penis in white and coloured men (100 white and 39 coloured) admitted to a Veterans' Hospital in Illinois. None of them had been circumcised before the age of 6. Comparison was made with control groups of men admitted to the same hospital for other affections. The incidence of venereal disease upon the various series was the same. The results (Table V) show that circumcision in the first six years was protective, while circumcision in later years (6–35) showed no significant difference in distribution between the various groups.

Lenowitz and Graham (1946) give further details of these cases from a Veterans' Hospital which enable one to compare these data with those of Dean

TABLE V.—*Circumcision in Patients with Carcinoma of the Penis and in Control Patients.* (Schrek and Lenowitz.)

| | Number of patients. | Percentage circumcised at age of— | | |
|---|---|---|---|---|
| | | 0–5 years. | 6–16 years. | 17–35 years. |
| *White.* | | | | |
| Men with carcinoma of penis | 100 | 0 | 2 | 5 |
| Controls | 188 | 12·8* | 2·1 | 3·7 |
| *Coloured.* | | | | |
| Men with carcinoma of penis | 39 | 0 | 0 | 3 |
| Controls | 168 | 17·9* | 3·0 | 3·6 |

* Difference from group with carcinoma of penis is significant.

and Lewis (Table IV). The averages of the two series are very similar, which suggests that the cases, though only 16 in number, provide a reasonable basis for study (Fig. 1). Circumcision at ages from 14 to 45 did not prevent the development of cancer after from 8 to 41 years, the mean interval being 23 years.

Cases in which the interval between circumcision and the diagnosis of cancer was less than the minimum (8 years) in Table IV have been omitted, in view of the possibility that cancer was already present at the time of the operation. Thus Lewis (1931) says of two of his cases, in which cancer was found 6 years, and 6 months, after the operation, that it was " entirely possible that both these patients had early malignancy at the time of the circumcision."

(d) *Cancer of the penis in Ceylon, Siam and China.*

This form of cancer is extremely frequent in some countries where circumcision is not practised and phimosis is common.* Spittel (1923), writing from Colombo, in Ceylon, says :" Carcinoma of the penis is another astonishingly common form of malignant disease in this country. Between June, 1911, and June, 1915, no fewer than 91 cases were operated on by me at the General Hospital, and I was but one of three surgeons. . . . Congenital phimosis is the almost invariable accompaniment."

Thomson (1921), in giving a statistical account of 13,761 operations of all kinds at the Canton Hospital, China, records 36 cases of cancer of the penis. In a private communication Dr. Thomson writes : " Cancer of the penis is relatively common. The patients are usually in their prime, and for the most part farmers without any venereal history. The condition is usually quite advanced when the patients come to the hospital and requires a radical operation." Bercovitz (1919) tabulates the records of 131 operations for cancer performed in Hainan, China ; of these operations, 29 were for cancer of the penis. Jefferys and Maxwell (1910), writing of cancer, other than that of the breast, in China, say, " The other frequent forms of carcinoma which present themselves for operation are of the lips and tongue, of the penis and of the rectum, of the uterus, and in Formosa, of the

* The importance of phimosis is of course attested also by much clinical evidence from Europe and America, e.g. in the series recorded by Dean (1935) only 4 out of 93 patients had a freely retractible prepuce. The only evidence to the contrary which I have found in the literature is that of Lewis (1931) in a report on 34 cases of carcinoma of the penis operated on at Johns Hopkins Hospital ; he says, " Nineteen of our series had retractible foreskins without a history of phimosis, five had acquired phimosis during the growth of the tumour, while nine gave a history of a long non-retractible foreskin." A retractible prepuce does not, of course, of itself ensure cleanliness.

mouth. . . ." They quote the words of another surgeon, McCartney, practising in China: "Epithelioma of the penis is a comparatively frequent disease, and we have made a complete amputation in a number of cases during the year . . ."

Noble (1943) records 52 cases of carcinoma of the penis (49 Siamese, 3 Chinese), with phimosis in 24 of these, in 231 admissions of males for carcinoma during 4 years at Bangkok, Siam.

Hence the available evidence is concordant in showing that cancer of the penis—

(i) is absent in those circumcised soon after birth ;
(ii) occurs in those circumcised from the 3rd to the 14th year ;
(iii) is very common in some countries of Asia where circumcision is not practised.

4. *Possible Modes of Action of Circumcision as a Preventive of Cancer.*

Cancer of the penis is thus a form of cancer which is dependent upon a single known factor, namely, the presence of a prepuce, for it is prevented altogether by removal of this factor at a sufficiently early age. "The prophylactic treatment of cancer of the penis consists in circumcising all male infants a few days after birth" (Dean, 1935). The postponement of the operation for 14 years only appears, on the evidence of the series of Dean, and of Lenowitz and Graham, to allow changes to occur which will later, after an interval of the order of 23 years, culminate in the development of cancer. The possible implications of this difference between early and later circumcision are so important that the evidence must be examined in detail.

(a) *Removal of the cancer-bearing area.*

One must, of course, consider the possibility that the immunity of circumcised persons from cancer of the penis is due to the removal of the area where such cancers would arise. But according to most authorities the prepuce is not the most common site of cancer. Purdy Stout (1932) says : "The vast majority of penile cancers are squamous-cell epithelioma, starting from the corona, sulcus or glans penis," *i.e.* from parts not removed in circumcision. Lederman (1941) says : "In general, it would seem that this frequency decreases in the following order—coronal sulcus, glans, prepuce and frenum ; but in an appreciable number of cases the disease when first seen is too advanced to allow of such refinement of observation." Barney (1907) says that "in 65 cases, where there was any definite statement on this point 45 . . . had started on the glans, while 24 had their origin on the prepuce. . . . Many had started in the sulcus behind the corona glandis . . ." [there is a slight arithmetical error here]. Ngai (1933) states that in 89 cases of cancer of the penis in Chinamen the tumour, according to the patient, arose in the following areas :

| | |
|---|---|
| Glans . . . . . . . | 39 |
| Prepuce . . . . . . | 37 |
| Coronary sulcus . . . . . | 11 |
| Frenulum and meatus . . . . | 1 each |
| | 89 cases |

Leighton (1932) in a study of 67 cases says: "In 30 of our cases the disease began on the glans penis and in 13 the first appearance was on the prepuce, while a few have thought that the growth began in the sulcus." Dean (1935) in his study of 120 cases (see above) says: "In this series nearly all the early lesions grew from the proximal third of the glans, the coronal sulcus, close to either side of the frenum, or from the mucosa of the prepuce in its proximal third." In the 34 operable cases recorded by Lewis (1931) the growth was said to have begun on the glans in 20, on the sulcus or corona in 6, and on the prepuce in 4.

Föderl (1926) gives the distribution in his series from the 2nd Surgical University Clinic in Vienna as follows:

| | | | |
|---|---|---|---|
| Sulcus coronalis | 12 | Frenulum | 3 |
| Glans | 11 | Orifice of Prepuce | 3 |
| Prepuce, internal surface | 6 | Orifice of Urethra | 3 |
| Prepuce, external surface | 1 | | |

Some other authorities, however, record a larger proportion of preputial growths. Lenowitz and Graham (1946) in their study of 139 cases at the Hines Veterans' Hospital, Illinois, class together growths on the frenum and prepuce and give the following figures for those in which the site of origin could be ascertained:

| | Frenum and prepuce. | Glans. | Corona. |
|---|---|---|---|
| White | 38 | 27 | 4 |
| Negro | 21 | 7 | 5 |
| Total | 59 | 34 | 9 |

Kaufmann (1886) states that out of his 33 cases, 29 began as a small wart, and of these 16 were on the prepuce and 13 on the glans. But a series of 29 cases is perhaps scarcely adequate for a statistical decision.

Thus, if one allows that as much as one-half of carcinomas of the penis arise on the prepuce, circumcision, if it acted solely by elimination of a potential cancer-bearing area, would lessen the incidence by one-half and would not account for the complete immunity of Jews. On this basis the number of cases to be expected in Sorsby's calculation (p. 336) would be 6 instead of 12.

(b) *Resistance of epithelium.*

Dean (1935) makes the following suggestion: "There must be some reason why circumcision in infancy immunizes against cancer of the penis while circumcision later in life does not. It may be that when an infant is circumcised and the glans is no longer protected by the prepuce, a denser, thicker epidermis develops, which is resistant to the formation of cancer by chronic irritation. When circumcision is performed in later years the glans may have lost its ability to produce a resistant covering, and although there is no longer irritation from retained secretions, the glans remains relatively sensitive to the contacts of everyday life."

(c) *External and internal agents.*

This suggestion brings one to the important question whether cancer of the penis is caused by a wholly external agent, in the same way as cancer of the scrotum can be caused by soot, or by an agent arising from the body, either between the prepuce and glans, or deep to the epidermis. A purely external agent appears unlikely, for a circumcised person is exposed, not less, but more, to such a factor, and the thickened epidermis supposed by Dean would have to compensate for such exposure ; moreover an unretracted prepuce, the usual antecedent of cancer, would be protective. The influence of phimosis in predisposing to cancer of the penis suggests that the carcinogenic agent is formed in material between the prepuce and glans.

### DISCUSSION.

Perhaps the most probable, and the most interesting, interpretation of the data given above is to suppose that the train of events leading to this malignant growth is set going in the earlier years of life, and that removal of the cause does not then avert the final appearance of cancer at a much later age. Thus, even if we ignore all the evidence from Moslem circumcision in view of any doubt about the completeness of the operation, the data collected by Dean at the Memorial Hospital, New York, and by Schrek and Lenowitz at a Veterans' Hospital in Illinois (Tables IV and V, Fig. 1), show that circumcision at the age of 14 and onwards may be followed by the development of cancer after an average interval of 23 years, while circumcision in infancy prevents this disease altogether. This may hold good of other forms of cancer also. Schrek and Lenowitz, in their study of cancer of the penis, suggest that " Perhaps other factors operating in infancy determine whether the adult develops other types of cancer." Thus cancer of, say, the stomach, arising after the second 25 years of life, may be predestined to occur by factors to which the body was exposed during the first 25 years after birth. This idea suggests interesting possibilities of the prophylaxis of cancer by attention to the hygiene of youth. The juvenile death-rate, as a measure of social factors, draws attention to the children who die, whereas those who do not die are, from the present point of view, more important ; a child may survive injury by social conditions of which the effect appears later in life.

The mean age at diagnosis of the 100 white men studied by Schrek and Lenowitz (Table V) was 50·4, while the corresponding age of the seven among them who had been circumcised was 43.* Admittedly these numbers are small for any comparison of averages, but the earlier age of the seven suggests that the operation had no protective action signalized by postponement of the disease. This indication is compatible with the idea suggested here that the occurrence of this form of cancer is determined at an early age. Possibly the earlier development of cancer in the circumcised men was due to the carcinogenic effect of phimosis, for which the operation was performed.

This phenomena of the appearance of cancer long after the cessation of exposure to a causal agent is, of course, known to occur in the case of industrial cancers, *e.g.* cancer of the scrotum may arise in a cotton spinner many years (from 1 to 14, Henry (1928)) after he has left the mule room. X-ray cancer provides a good instance of the same process and is of peculiar value, because the cessation of the primary action is abrupt, whereas chemical agents may persist for an unknown length of time in the skin. But the instance of cancer of the penis is of more interest, first, because no artificial agents are concerned, and second, because the essential changes take place in the early years of life.

* No comparison is possible with the age of maximum incidence in the general population, which is later, as the Veterans are a group selected as regards age.

### SUMMARY.

1. Cancer of the penis does not occur after circumcision on the eighth day according to the Jewish practice, but occurs in later life in Moslem populations, where the operation is carried out between the 3rd and 14th years. But no record has been found in the literature of the age-relations in even a single case of cancer of the penis in a Moslem.

2. Sixteen recorded cases of cancer of the penis following surgical circumcision at a mean age of 23 (range 14 to 45) developed cancer of the penis after a mean interval of 23 years (range 8 to 41 years).

3. The failure of the operation deferred until the 14th year to give the protection given by it when carried out on the 8th day suggests that the train of events leading to the malignant growth is set going early in life, and that removal of the cause does not then avert the development of cancer at a much later age. Other forms of cancer are perhaps due to factors acting in youth.

4. Cancer of the penis is very prevalent among some peoples of Asia who do not practise circumcision.

5. The protection given by the Jewish operation is not due to removal of the cancer-bearing area.

### REFERENCES.

Anderson, W. S.—(1918) *Sth. med. J.*, **11,** 448.
Barney, J. D.—(1907) *Ann. Surg.*, **46**, 890.
Bercovitz, N.—(1919) *J. Cancer Res.*, **4**, 229.
Dean, A. L.—(1935) *J. Urol.*, **33**, 252.—(1936) *Trans. Amer. Ass. gen.-urin. Surg.*, 493.
Föderl, V.—(1926) *Dtsch. Z. Chir.*, **198,** 207.
Henry, S. A.—(1928) *J. Hyg.*, **28**, 100.
Jefferys, W. H., and Maxwell, J. L.—(1910) 'Diseases of China, including Formosa and Korea,' Philadelphia and London.
Kaufmann, C.—(1886) *Dtsch. Z. Chir.*, **50***a*, 266.
Kouwenaar, W.—(1933) *Geneesk. Tijdschr. Ned.-Ind.*, 1539.
Kretschmer, H. L.—(1918) *Surg. Clin. Chicago*, **2**, 269.
Lane, E. W.—(1842) 'Manners and Customs of the Modern Egyptians,' 3rd edn.
Lederman, M.—(1941) *Post grad. med., J.*, n.s., **17**, 97.
Leighton, W. E.—(1932) *Amer. J. Cancer*, **16**, 251.
Lenowitz, H., and Graham, A. P.—(1946) *J. Urol.*, **56,** 458.
Lewis, L. G.—(1931) *Ibid.*, **26**, 295.
Megaw, J. W. D.—(1905) *Indian med. Gaz.*, **40**, 163.
Nath, V., and Grewal, K. S.—(1935) *Indian J. med. Res.*, **23**, 149.
Ngai, S. K.—(1933) *Amer. J. Cancer*, **19**, 259.
Noble, T. P.—(1943) *Brit. J. Urol.*, **5**, 243.
Preuss, J.—(1923) *Biblisch-talmudische Medizin, Berlin.*
Schrek, R., and Lenowitz, H.—(1947) *Cancer Res.*, **7**, 180.
Sorsby, M.—(1931) 'Cancer and Race,' London.
Spittel, R. L.—(1923) *Brit. med. J.*, **2**, 632.
Stout, Purdy.—(1932) 'Human Cancer,' London.
Sutherland, D. W.—(1904) *Arch. Middlesex Hosp.*, **3**, 84.
Thomson, J. O.—(1921) *Ann. Surg.*, **73**, 217.
Wallace, A. R.—(1898) 'The Malay Archipelago,' London, 3rd ed., Chap. VII.
Wolbarst, A. L.—(1932) *Lancet*, i, 150.
Wolff, G.—(1939) *Amer. J. Hyg.*, **29**, 121.